# Double Trouble: A Primary Epstein-Barr Virus Infection Causing Cholestatic Hepatitis and Hemophagocytic Lymphohistiocytosis

**DOI:** 10.7759/cureus.31014

**Published:** 2022-11-02

**Authors:** Joanne Lin, Geetha Sivasubramanian

**Affiliations:** 1 Internal Medicine, University of California San Francisco, Fresno, USA; 2 Infectious Disease, University of California San Francisco, Fresno, USA

**Keywords:** ebv hlh, young adult male, ebv- associated hepatitis, cholestatic liver injury, hemophagocytic lymphohistiocytosis (hlh), primary ebv infection

## Abstract

Primary infection with Epstein-Barr virus (EBV) is very common, often manifesting as mononucleosis syndrome with fatigue, sore throat, fever, and enlarged lymph nodes. Liver involvement occurs in many cases with mildly elevated liver enzymes. However, it is rare to see EBV infection present as cholestatic hepatitis. Another rare complication of primary EBV infection is hemophagocytic lymphohistiocytosis (HLH). We describe a patient with primary EBV infection who presented with fatigue and jaundice, subsequent rash, and reactive lymphocytosis. The patient was noted to have cholestatic hepatitis and was highly suspected to have HLH based on laboratory values, including elevated ferritin, triglyceride, and interleukin-2 levels. He showed clinical improvement with HLH treatment using dexamethasone, etoposide, and rituximab. We further review the clinical manifestations, pathogenesis, and management of EBV-associated cholestatic hepatitis and EBV-HLH. Early diagnosis of primary EBV infection is emphasized in order to properly recognize and treat potentially life-threatening complications.

## Introduction

Epstein-Barr virus (EBV), also known as human herpesvirus 4, is one of the most ubiquitous human viruses. It is estimated that more than 90% of the world’s population is seropositive to EBV [1]. Transmission is usually through oropharyngeal secretions and primary infection of the virus is the causative agent of infectious mononucleosis, which classically presents with the triad of fever, pharyngitis, and lymphadenopathy [2]. Although many primary EBV cases are asymptomatic or present with lymphocytosis, mild thrombocytopenia, and rash, primary EBV infection amongst adolescents and adults can manifest with varying clinical presentations and severity. Hepatic involvement is common in primary EBV infections, involving about 80-90% of cases, and usually causes a transient mild to moderate increase in transaminases [3]. However, clinical manifestations of cholestatic hepatitis, such as marked icterus and jaundice, are infrequent with an incidence of less than 5% [4]. Moreover, hemophagocytic lymphohistiocytosis (HLH) is a rare life-threatening complication of primary EBV infection that usually occurs in immunodeficient individuals, such as those with X-linked lymphoproliferative (XLP) disorder. We report a case of EBV-induced acute cholestatic hepatitis and HLH in an immunocompetent adult who presented with fever, malaise, and jaundice.

## Case presentation

A 38-year-old healthy Hispanic man presented to the emergency room with a four-day history of fever, jaundice, fatigue, and generalized weakness. The patient denied nausea, vomiting, abdominal pain, chest pain, shortness of breath, dysuria, and diarrhea. He denied taking any medications or supplements, family history of liver or autoimmune diseases and malignancy, intravenous drug use, daily or extensive alcohol use, recent travel, or sick contacts. On initial presentation, the patient was febrile with a max temperature of 38.4C and mildly tachycardic with a heart rate of 109 per minute. Positive exam findings included scleral icterus and diffuse jaundice. There was no visible rash, lymphadenopathy, or evidence of pharyngitis noted. The rest of his cardiovascular, respiratory, and abdominal examinations were unremarkable. He later developed a new diffuse maculopapular rash during his hospitalization.

Laboratory values revealed a cholestatic hepatitis pattern (Table 1).

**Table 1:** Laboratory values revealing a cholestatic hepatitis pattern.

Laboratory test	Results	Normal range
Alkaline phosphatase (ALP)	173 U/L	25-100 U/L
Alanine aminotransferase (ALT)	106 U/L	10-40 U/L
Aspartate aminotransferase (AST)	196 U/L	8-40 U/L
Total bilirubin	27.9 mg/dl	0.2-1.2 mg/dl
Direct bilirubin	22 mg/dl	< or =0.2 mg/dl

**Figure 1:**
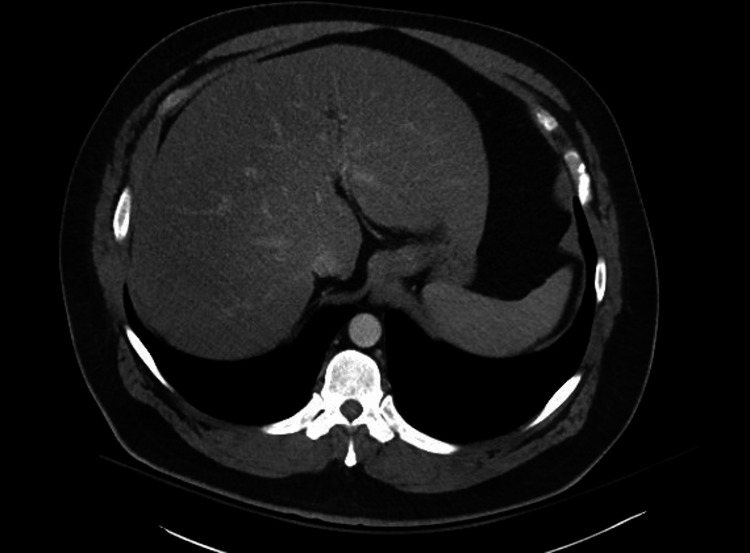
Computed tomography (CT) abdomen/pelvis showing fatty liver with no obstruction, masses, or stones.

**Figure 2:**
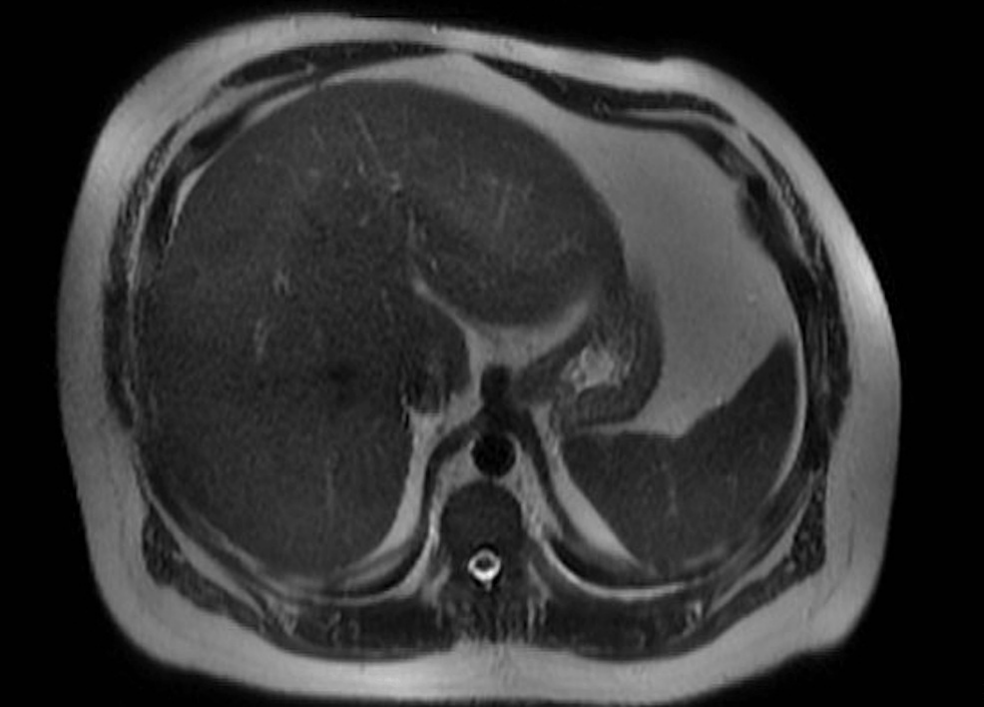
Magnetic resonance cholangiopancreatography (MRCP) showing no obstruction, stones, and ductal dilation.

The patient also had a lymphocytic leukocytosis of 18.7 x 10*3 U/L (normal 4-11 x 10*3 U/L) with a reactive lymphocytosis of 3% (normal 0-1%) and anemia with hemoglobin of 10 g/dL (normal 14-18 g/dL). Protein, albumin, PT/INR, and platelets were within normal limits. Imaging included CT scans of the abdomen and pelvis region which showed fatty liver with no obstruction, masses, or stones (Figure 1), and magnetic resonance cholangiopancreatography (MRCP) was unremarkable for obstruction, stones, and ductal dilation (Figure 2).

Further laboratory workup revealed the following, noted in Table 2.

**Table 2:** Patient’s further laboratory workup results.

Laboratory test	Results
Blood cultures	Negative
Mitochondrial antibody	Negative
Smooth muscle antibody	Negative
Hepatitis A antibody IgM	Negative
Hepatitis B surface antigen	Negative
Hepatitis B core antibody IgM	Negative
Hepatitis C antibody	Negative
HIV screen	Negative
Rapid plasma reagin (RPR)	Negative
Brucella	Negative
Toxoplasma	Negative
Cocci antibody	Negative
Cytomegalovirus	Negative
G6PD	Negative
ANA titer	1:80
Ceruloplasmin	56 mg/dL (normal 18-36 mg/dL)
Erythrocyte sedimentation rate (ESR)	40 mm (normal 0-15 mm)
C-reactive protein (CRP)	146.3 mg/L (normal 0-3 mg/L)
Ferritin	>16,500 ng/mL (normal 22-322 ng/mL)
Lactate dehydrogenase (LDH)	1676 U/L (normal 100-230 U/L)
Triglycerides	380 mg/dL (normal 30-159 mg/dL)
Interleukin 2 receptor level	1014.6 pg/ml (normal 175.3-858.8 pg/ml)

**Figure 3:**
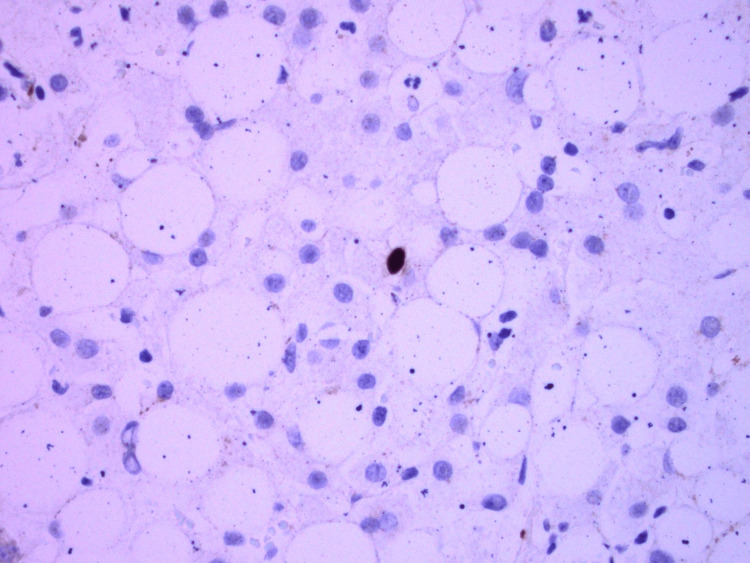
Liver biopsy showing single brown staining nuclei in the center positive for Epstein-Barr virus.

**Figure 4:**
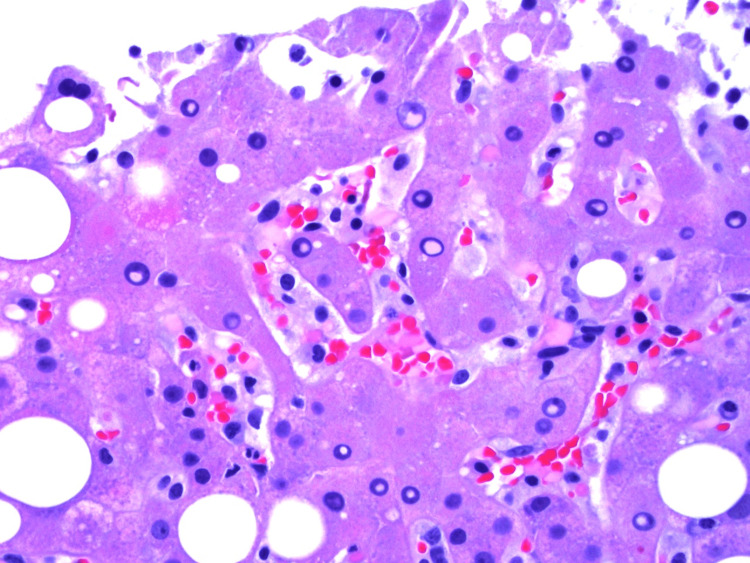
Liver biopsy showing hemophagocytosis in sinusoids.

**Figure 5:**
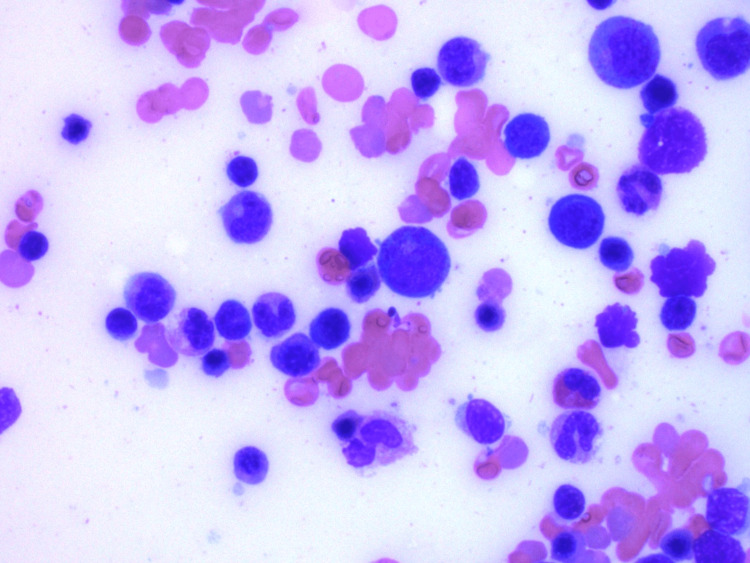
Bone marrow biopsy showing mixed elements (erythroid, myeloid, lymphoid).

EBV nuclear antigen (EBNA) was negative, but EBV DNA polymerase chain reaction (PCR) eventually resulted as positive with 10,347 copies/mL (normal <200 copies/mL). A liver biopsy showed EBV viral hepatitis with positive EBER stain, steatohepatitis, and hemophagocytosis (Figures 3-4), while a bone marrow biopsy showed hypercellular marrow without a prominent infiltrate of histiocytes (Figure 5). Natural killer (NK) cell activity and X-linked lymphoproliferative (XLP) genetic testing (SH2D1A mutation) were negative.

The patient was initially started on ampicillin-sulbactam for concern of cholangitis, but antibiotics were discontinued after reviewing the findings of the MRCP. Treatment of HLH was initiated with dexamethasone, etoposide, and rituximab. The patient’s laboratory values including total bilirubin, alanine aminotransferase (ALT), aspartate aminotransferase (AST), ferritin, and lactate dehydrogenase (LDH) continued to downtrend with treatment. He was eventually discharged home with close primary care and hematology follow-up.

## Discussion

Although EBV is one of the most common viruses globally, its nature of variable clinical manifestations as a primary infection often makes it an elusive diagnosis. Patients usually present with the triad of fever, pharyngitis, and lymphadenopathy. In our case, our patient only had fever and fatigue without pharyngitis or lymphadenopathy. Diagnosing EBV correctly requires an understanding of the blood antigen and antibody serology. An acute EBV infection is indicated by a positive anti-viral capsid antigen (VCA) IgM and IgG with a negative EBNA. A recent infection within the last 3-12 months is determined by positive anti-VCA IgG and EBNA, negative anti-VCA IgM and usually positive anti-early antigen (EA) antibodies [5]. In this case, our patient's EBNA was negative, but EBV DNA PCR was positive which confirmed an active acute viral replication. EBV anti-VCA IgM and IgG were not sent for. 

Hepatic involvement is common with primary EBV infection, especially as the age of the patient increases, but it usually presents as mild ALT and AST elevation, less than a five-fold increase from the normal range [6]. A study following 41 immunocompetent patients diagnosed with hepatic dysfunction associated with acute primary EBV infection demonstrated ALT and AST levels had several peaks, returning to normal range on average 20 days after symptom onset; many patients ranged between the one- to five-fold increase mark [3]. Our patient had hepatic involvement and his laboratory values fell between the one- to five-fold increase in transaminase levels similar to the patients in the study. However, he also had persistently elevated total bilirubin, which is noted by the study to be uncommon, as most patients do not present with a cholestatic pattern defined as predominantly elevated serum alkaline phosphatase and bilirubin levels. The study showed that bilirubin levels tend to be only mildly elevated and observed in 20% of the patients [3]. Given the broad differentials of cholestatic hepatitis, work-up for cholestasis is important and first involves determining if the cholestasis is intra- or extrahepatic with imaging in addition to laboratory evidence. It is important to rule out extrahepatic causes with ultrasound and more specific imaging such as MRCP if necessary. Intrahepatic causes of cholestatic hepatitis also need to be considered, including viral infections such as viral hepatitis, EBV, cytomegalovirus, HIV, medications or herbal supplements, primary biliary cholangitis, and primary sclerosing cholangitis. The pathogenesis of cholestasis in EBV infection remains unclear but currently is thought to be secondary to an immune-mediated response during which there is inflammation and damage of the bile ducts or direct hepatic cellular damage by the autoantibody-mediated activation of free radicals [4]. The management of EBV cholestatic hepatitis consists of supportive symptomatic management with antipyretics and analgesics with a trial of cholestyramine for persistent pruritus.

HLH is a severe inflammatory syndrome associated with a dysregulated immune response to malignancies or infections. The pathogenesis of EBV-HLH is due to the activation of cytotoxic T lymphocytes by EBV-infected B cells, leading to hypercytokinemia and the stimulation of histolytic cells. Moreover, EBV itself causes unregulated production and secretion of interleukins, INFa, T- and NK- cells [7]. EBV-HLH initially can mimic primary EBV infection with a presentation of fever, hepatosplenomegaly, and hepatitis. Along with the clinical presentation, at least five of eight criteria must be fulfilled to diagnose HLH unless a molecular diagnosis can be confirmed (Table 3).

**Table 3:** Diagnostic criteria for hemophagocytic lymphohistiocytosis NK cells: NK killer cells

Diagnostic Criteria for Hemophagocytic Lymphohistiocytosis
Fever (temperature > 38.5 C)
Cytopenia affecting at least 2 cell lines: hemoglobin less than 9 g/L, platelets less than 100 x10^9^/L, neutrophils less than 1 x10^9^/L
Hypertriglyceridemia (>265 mg/dL) and/or hypofibrinogenemia (<150 mg/dL)
Splenomegaly
Hemophagocytosis in bone marrow, spleen, or lymph node
Low or absent NK cell activity
Elevated serum ferritin (>500 ug/L)
Elevated soluble CD25 or interleukin-2 receptor level (>2400 U/mL)

However, if HLH is highly suspected, treatment should not be delayed while awaiting test results or failure to meet five of the eight diagnostic criteria, as EBV-HLH is a life-threatening condition requiring early treatment with immunosuppressive therapy [8]. EBV-HLH treatment involves prompt initiation of dexamethasone, etoposide, and rituximab [9]. In addition, although rare, genetic disorders such as XLP disorder should be considered, particularly when male patients have a presentation of severe liver damage or failure, aplastic anemia, hypogammaglobulinemia, suspicion of B-cell lymphoma, or HLH [10]. The lack of proper immune cell development and maturation in XLP patients makes these patients susceptible to a fatal course of EBV infection [11].

## Conclusions

Primary EBV infection can manifest with unique presentations of symptoms and laboratory results. Clinicians should consider early testing for EBV with anti-VCA IgM or EBV DNA PCR in patients who present with rash, cholestatic hepatitis, and reactive lymphocytosis. Primary EBV infection has a high incidence of hepatic involvement and can also cause life-threatening complications such as HLH. Timely diagnosis and treatment are crucial to reducing morbidity and mortality.

## References

[1] Smatti MK, Al-Sadeq DW, Ali NH, Pintus G, Abou-Saleh H, Nasrallah GK (2018). Epstein-Barr virus epidemiology, serology, and genetic variability of LMP-1 oncogene among healthy population: an update. Front Oncol.

[2] Mohseni M, Boniface MP, Graham C (2022). Mononucleosis. https://www.ncbi.nlm.nih.gov/books/NBK470387/.

[3] Kofteridis DP, Koulentaki M, Valachis A, Christofaki M, Mazokopakis E, Papazoglou G, Samonis G (2011). Epstein Barr virus hepatitis. Eur J Intern Med.

[4] Khoo A (2016). Acute cholestatic hepatitis induced by Epstein-Barr virus infection in an adult: a case report. J Med Case Rep.

[5] Kang MJ, Kim TH, Shim KN, Jung SA, Cho MS, Yoo K, Chung KW (2009). Infectious mononucleosis hepatitis in young adults: two case reports. Korean J Intern Med.

[6] Alli A, Nabil F, Ortiz JF (2021). Infectious mononucleosis: a case report with unusual features and abnormal laboratory findings. Cureus.

[7] Goudarzipour K, Kajiyazdi M, Mahdaviyani A (2013). Epstein-Barr virus-induced hemophagocytic lymphohistiocytosis. Int J Hematol Oncol Stem Cell Res.

[8] Henter JI, Horne A, Aricó M (2007). HLH-2004: Diagnostic and therapeutic guidelines for hemophagocytic lymphohistiocytosis. Pediatr Blood Cancer.

[9] Jordan MB, Allen CE, Weitzman S, Filipovich AH, McClain KL (2011). How I treat hemophagocytic lymphohistiocytosis. Blood.

[10] Marsh RA (2017). Epstein-Barr virus and hemophagocytic lymphohistiocytosis. Front Immunol.

[11] (2022). X linked lymphoproliferative syndrome. https://rarediseases.org/rare-diseases/x-linked-lymphoproliferative-syndrome/.

